# How *Naunyn-Schmiedeberg’s Archives of Pharmacology* deals with fraudulent papers from paper mills

**DOI:** 10.1007/s00210-021-02056-8

**Published:** 2021-02-06

**Authors:** Roland Seifert

**Affiliations:** Institute of Pharmacology, Hannover Medical School, Carl-Neuberg-Str. 1, D-30625 Hannover, Germany

**Keywords:** Paper mills, Scientific fraud, Scientific integrity, Fake data, Raw data

## Abstract

Fraudulent papers from paper mills are a serious threat to the entire scientific community. *Naunyn-Schmiedeberg’s Archives of Pharmacology* has become the target of a massive attack of fraudulent papers originating from paper mills. This editorial highlights 20 important features we observed with paper mills and explains how the journal is responding to this serious threat to restore the integrity of science. Hopefully, this editorial is also helpful for editors of other scientific journals.

Paper mills are commercially motivated malicious enterprises apparently operated by knowledgeable “scientists” that produce custom-designed papers containing no real scientific data but only fake data. Depending on the amount of money the customer is willing to pay, paper mills also offer manuscript submission and manuscript revision services and take care of all correspondence with the journal (full-service premium package). The purpose of paper mills is to promote the “academic career” of their customers by delivering publications in prestigious scientific journals.

Two recent excellent papers have summarized several major (sad) features of paper mills (Byrne and Christopher 2020; Miyakawa 2020). Various science blog sites discuss in detail the background and commercial motivations of paper mills (see, e.g., https://forbetterscience.com/2020/01/24/the-full-service-paper-mill-and-its-chinese-customers/).

Unfortunately, *Naunyn-Schmiedeberg’s Archives of Pharmacology* became a victim of paper mills. The Editor-in-Chief was alerted of the problem by science blog sites in February 2020 and responded immediately at various levels by posting statements on blog sites and informing associate editors, the publisher SpringerNature, other scientific journals and colleagues. The paper mill crisis was also intensively discussed at the Editorial Board meeting of the journal in March 2020 in Leipzig, Germany. Most importantly, the Editor-in-Chief immediately contacted the authors of suspected fraudulent papers. The paper mill attack on the journal has resulted in several retractions of papers. The first five corresponding retraction notes are published in this issue (Table 1), and we expect five more retractions to be completed in 2021.

**Table 1:** List of the retraction notes of fraudulent papers published in the March 2021 issue *Naunyn-Schmiedeberg’s Archives of Pharmacology*

Retraction Note to: Resibufogenin suppresses tumor growth and inhibits glycolysis in ovarian cancer by modulating PIM1 Qian Li, Chuanwu Jiang , Yan Wang, Minghua Wei, Huijin Zheng, Yanqi Xu, Xuegang Xu, Fengyu Jia, Kai Liu, Gang Sun, Jianhua Zang, and Ping Mo. Naunyn-Schmiedeberg's Archives of Pharmacology. 10.1007/s00210-020-02016-8	
Retraction Note to: Fibrauretine reduces ischemia/reperfusion injury via RISK/eNOS activation Chunsheng Wang, Rong Chang, Gan Gao, Xing Liu and Yingwei Zhang. Naunyn-Schmiedeberg's Archives of Pharmacology. 10.1007/s00210-020-02027-5	
Retraction Note to: Soyasapogenol B exhibits anti-growth and anti-metastatic activities in clear cell renal cell carcinoma Luping Wang, Junyu Wang, Hong Zhao, Guoping Jiang, Xiaojie Feng, Wenxia Sui and Hongling Liu. Naunyn-Schmiedeberg's Archives of Pharmacology. 10.1007/s00210-020-02020-y	
Retraction Note to: Chrysophanol suppresses growth and metastasis of T cell acute lymphoblastic leukemia via miR-9/PD-L1 axis Junjie Yin, Qingsong Yin, Bo Liang, Ruihua Mi, Hao Ai, Lin Chen and Xudong Wei. Naunyn-Schmiedeberg's Archives of Pharmacology. 10.1007/s00210-020-02026-6	
Retraction Note to: Chrysophanol exhibits anti-cancer activities in lung cancer cell through regulating ROS/HIF-1a/VEGF signaling pathway Jie Zhang, Qian Wang, Qiang Wang, Peng Guo, Yong Wang, Yuqing Xing, Mengmeng Zhang, Fujun Liu and Qingyun Zeng. Naunyn-Schmiedeberg's Archives of Pharmacology 10.1007/s00210-020-02019-5	

We have observed 20 important features among papers originating from paper mills:

1. Commercial email addresses: In all papers, just commercial email addresses were provided. Never academic email addresses were provided. Often, the email addresses had little relation to the name of the corresponding author. In several cases, authors claimed that their institutions do not provide them with academic email addresses. Evidently, the lack of use of academic email addresses renders identification of the fraud authors much more difficult, if not impossible. In the meantime, we have learned that all “real” academic institutions across the world provide academic email addresses. It has just become a bad global habit that many scientists use commercial email addresses for convenience, and this security gap is aggressively exploited by paper mills. Therefore, our journal does not allow anymore submissions without a valid academic (or pharmaceutical company) email address.
2. Uneventful peer review process: All papers passed the routine text similarity check with the iThenticate software without any problem. With very few exceptions, the peer review of papers was very uneventful and went smoothly. Editors handled paper mill papers without noting anything unusual. In this context, it must be noted that we do have an excellent reporting system in place should anything unusual happen during peer review. These safeguards were bypassed effectively by the paper mills. This is indicative of the “professionalism” behind paper mills. It can be assumed that experienced “scientists” (or more precisely misguided ex-scientists) with extensive and in-depth experience in the scientific publication process and familiarity with professional plagiarism software are the core of paper mills. It is likely that employees of paper mills have worked with real scientific journals before. Consequently, in our revised editorial guidelines, we request an explicit statement by authors that no paper mill was used.
3. “Scientific” focus of the fraud: Most paper mill papers in our journal deal with chemically defined natural compounds, mostly from plants in the context of “highly important,” “highly relevant,” or “medically underserved” diseases for which there is “no cure yet” and a “high medical need.” Often, paper mill papers advertise a breakthrough in a given pharmacological field. Thus, it can be assumed that customers are not just interested in getting “a” publication but a “medically important” publication. This is compatible with the assumption that the primary purpose of paper mills is to facilitate rapid “academic career” advancement and professional rewards for the “customer.” Since most paper mill papers allude to high clinical relevance, it can be assumed that “premium” customers of paper mills are clinicians who have no scientific experience. This assumption is corroborated by the fact that in many cases, authors list clinical departments as their affiliation.
4. No reporting of service laboratories: Several papers included data from so-called service laboratories, but the papers did not mention this fact in the Materials and Methods section. Therefore, in our revised editorial guidelines, we request an explicit statement that all data were generated in-house.
5. Elusive “service laboratories”: Authors blame the “service laboratories” of having provided “problematic data,” but we never obtained any information about the precise nature of the “problematic data.” In no case, the authors admitted that the data from “service laboratories” are fake, and not a single service laboratory could be localized physically (postal address or internet address). In no case, the name of a person behind a “service laboratory” was provided. Thus, it appears that paper mills do their very best to prevent identification of the “service laboratories” because they are an integral part for their business to be successful.
6. No data at all: In all cases, the authors were unable to provide original data (raw data). Sometimes no reason was given; sometimes ridiculous reasons were given (the COVID-19 pandemia being the most popular “excuse”). The pandemia was blamed to have caused lack of access to data files or complete loss of data. This is a hitherto unknown facet of the pandemia that SARS-CoV-2 also infects computer hard disks and USB sticks. In an extreme case, an author’s child was blamed of having spilled coffee into the computer, resulting in total data loss with no back-up of data, “unfortunately.” Thus, in addition to having no data, the authors of paper mill papers have no clue how to properly store and back-up data. Because of the lack of original data in all fraud cases, in our revised editorial guidelines, we request original data already upon initial submission. Otherwise, no peer review will take place. The request of original data right from the beginning is the most effective measure to fight paper mills.
7. Cut and paste beautiful images: The fraud data particularly concerned flow cytometry experiments, fluorescence cell images, western blots, and histology. We noted several types of fraud: images were flipped. The same data (particularly flow cytometry panels, fluorescence cell images, and western blot bands) were used in experimentally totally different contexts, not just within one paper but also within completely different papers unrelated to each other. Most notably, in western blots, only “regions of interest” without molecular mass markers were shown, never full-length blots. Thus, it appears that paper mills arbitrarily collaged “impressive” data sets from a library of “data modules.” As a precaution against fraud of this type, in our revised editorial guidelines, we now request submission of full-length western blots with molecular mass markers. This is also a very effective measure to fight paper mills.
8. No email signatures: In no case, the corresponding authors used professional (institutional) email signatures in the emails. Apparently, an important element of paper mills is disguising the precise origin of the email and rendering it virtually impossible to localize the fraudulent author. Evidently, this substantially reduces the risk of negative academic consequences for the author.
9. Extremely poor English in emails: The English language used in email correspondence was extremely poor and, astonishingly, much worse than in the respective papers. In the papers, the English, in general, was decent and passed the English language check. This discrepancy indicates that the paper mill employees “polishing” papers (i.e., their final product) are much more proficient in English than the employees taking care of the journal correspondence that will not be published.
10. Rapid informal agreement to retraction: Most surprisingly, when confronted with the suspected fraud, quite often the corresponding authors very quickly (within very few hours and almost simultaneously!) agreed to a retraction without actually admitting the fraud openly. Often, authors mentioned the global term “problem with the data” without being specific what the problem is.
11. Blaming others: In some cases, corresponding authors blamed one of their graduate students (unnamed) of having generated “problematic” data, and that the respective student had already faced consequences (“punishment”) by the academic institution. In no case, we received any official evidence that a student was “punished” and what the precise academic misconduct and “punishment” was. Evidently, the corresponding authors do not recognize that they also bear a major responsibility for the entire group.
12. Painful formal retraction process: The formal retraction process was very sluggish from the side of the authors and lacked professional conduct.
13. Communication without content: Evasiveness and disinformation tactics prevailed in email communication with the corresponding authors. Often, the content of the emails was close to zero when it came to answering specific questions of the Editor-in-Chief or the publisher SpringerNature. Apparently, the authors wished to convey the impression that their English is “unfortunately” too poor to understand the questions of the Editor-in-Chief or the publisher.
14. Puzzling email clusters: Strikingly, in several fraud cases, the Editor-in-Chief received emails from authors from apparently different groups within a short period of time (minutes to hours!) as if the emails were sent by one and the same person under different email addresses. It was also noted that the text was rather similar. Thus, it can be assumed that a single paper mill employee is handling multiple fake submissions in one and the same “work session.”
15. Sluggish email communication (if any): Sometimes, there was no communication at all, because email addresses (especially of co-authors) did not work or because authors did not respond. Sometimes, we had to wait for up to 6 (!) months to get an answer. Blocking of emails by servers was used as another common “excuse” for not responding quickly. However, we never received any message from email servers that a message could not be delivered, unless an email address was invalid. The sluggish email communication is the major reason why it took so long to publish the retraction notes. We would have liked to proceed much faster, but we could not because the Editor-in-Chief and SpringerNature had to play the formal retraction process by the formal rules. Most unfortunately and to our dismay, fraudulent authors only care about rapid publication of their papers but not about rapid procedurally correct retraction. This is highly unethical and unprofessional conduct.
16. No academic institutions: In two cases, it turned out to be impossible to get into contact with the respective academic institutions from which the papers were generated.
17. No ORCID IDs: Very few authors used ORCID IDs. This is an effective method of disguising the identity of an author, particularly when first names and family names are very common in the literature.
18. Fake reviewers: When Editors used reviewers proposed by authors in fraud cases, in some cases, reviews were delivered in unusually short time (minutes!). In general, these reviews were very brief, uninformative, and written in extremely poor English. In all cases, these “fake reviewers” were not used as first-choice referees by the editors but as “very last-choice” when multiple attempts (up to 10) to recruit trusted referees failed. In this context, it must be noted that several of our trusted referees got extremely exhausted by the large number of review requests and, accordingly and understandably, declined. In fact, we received several complaints by referees for the high workload. We can only apologize to our referees. We were simply not aware of the problem. Thus, paper mill authors intentionally exploit and exhaust honest and trusted referees as a most valuable resource of a scientific journal so that desperate editors, as last-resort, use the author-suggested fake reviewers.
19. Same paper, different authors: In one particularly egregious case, a paper administratively withdrawn from our files because of suspected fraud was submitted shortly thereafter to another pharmacological journal, the only major difference being that the list of authors was totally different. The title of the paper was slightly modified (fraudulent submission NSAP-D-20-00138; authors Ning Li, Yan Wang, Wensheng Li, Haiyan Li, Liu Yang, Jun Wang: Aldosterone receptor antagonist**s**-mediated cognitive improvement in a mouse model of Alzheimer’s type: A key role of BDNF-H_2_S-Nrf2 signaling versus (Chen et al. 2020: Mineralocorticoid receptor antagonist-mediated cognitive improvement in a mouse model of Alzheimer’s type: possible involvement of BDNF-H_2_S-Nrf2 signaling). Changing the term “aldosterone receptor “against “mineralocorticoid receptor” points to the paper mill employees having solid pharmacological knowledge. Thus, it can be assumed that the paper declined in our journal was sold *de novo* to new “authors” without informing them that this paper has been flagged as fake already before. When the editor of *Fundamental & Clinical Pharmacology* was contacted by us, we were notified that the paper underwent regular (uneventful) peer review and that there is no need for action. We had the impression that the editor of *Fundamental & Clinical Pharmacology* was not aware of the severity of the problem. It is very likely that this example is not an isolated case because there is no generally accessible data base of rejected fake papers. Thus, paper mills can aggressively exploit this lack of data base on rejected fake papers and the fundamental assumption of author honesty for their business model. This type of fraud can only be detected by chance. As a minimalistic precaution against detection of this type of fraud, paper mills slightly change the paper title, barely disguising the identity of the content.
20. Geographical origin of fake papers: The retraction notes published in this issue (Table 1) and the additionally forthcoming retraction notes are all from one country. Thus, one can assume that paper mills reside in this country and that there must be substantial career-advancing incentives for “authors” to invest (private?) financial resources into paper mill papers. Interestingly, paper mills aim at disguising the geographical origin of the emails by sending them at any day or night time, strikingly deviating from “conventional” email writing and sending patterns by scientists from different continents. In addition, the risk of being uncovered is apparently deemed to be rather low by the fake authors and largely outweighed by the professional “benefits.” It must be assumed that a specific political climate in this country strongly fosters the use of paper mills. It cannot be excluded that paper mills operate in other countries as well. Therefore, in our revised editorial guidelines, the request for original data concerns authors from all countries. We deeply regret that we had to implement this rule globally, but we did not want to convey the impression of discrimination of a specific country.

All the above points show that the “success” of paper mills does not rely on a single tactic but a well-balanced portfolio of malicious strategies. These strategies complement each other to lead to the desired result. At the same time, the portfolio of strategies minimizes the risk of the fraud authors to be identified, minimizing the risk of negative professional consequences.

Once the Editor-in-Chief of *Naunyn-Schmiedeberg’s Archives of Pharmacology* had become aware of the problem and began to understand the tactics of paper mills, he immediately stopped online publication of all accepted papers. Only authors providing original data as electronic supplement to their papers were allowed to proceed to online and final publications. This first emergency measure prevented the online and final publication of another 10 fraudulent papers. Moreover, the Editor-in-Chief immediately interrupted the peer review process of all papers under consideration. Peer review was only allowed to continue when authors provided raw data. This second emergency intervention resulted in the drop-out of another 30 papers from the peer review and publication pipeline. In virtually all cases, authors never responded to the request of original data and ignored up to 10 (!) reminders. The respective papers were then all administratively withdrawn by the Editor-in-Chief prior to acceptance or rejection. All authors from fraudulent papers were flagged to prevent future submissions to the journal.

A total number of 10 paper retractions in a single journal within 1 year is quite a large. Even more dramatic, the high number of submissions (40) from paper mills caught prior to online publication illustrates that the problem was massive and that *Naunyn-Schmiedeberg’s Archives of Pharmacology* was considered to be a highly desirable and attractive target for “academic promotion,” both by customers and paper mills. With an average of about 1,000 submissions per year to our journal in 2019 and 2020, we come to the sad conclusion that around 5% of the submissions to our journal were from paper mills. Probably, this high percentage is related to the fact that the journal traditionally publishes many papers on natural compound pharmacology and that career incentives to publish in this field in the target country and in this journal must be very substantial.

If the Editor-in-Chief had not reacted immediately, *Naunyn-Schmiedeberg’s Archives of Pharmacology* probably would have experienced a further dramatic increase in paper mill submissions. Paper mills could have advertised the journal as “easy prey,” charging higher prices for customers for higher publication success rates. We may have stopped the fraud wave at the beginning of an exponential growth phase.

Quantitative comparisons of the fraud extent with other journals are impossible because of the lack of data, but at least one journal (*Molecular Brain*) in a somewhat different scientific field reports a substantial quantitative problem with fake submissions as well (Miyakawa 2020). Therefore, the Editor-in-Chief of *Naunyn-Schmiedeberg’s Archives of Pharmacology* believes that other journals in other areas may have become victim of paper mills as well. Lists of journals tentatively affected by paper mills posted on science blog sites corroborate the assumption that the problem does not only concern the field of natural compound pharmacology.

Following the emergency measures implemented in February 2020, *Naunyn-Schmiedeberg’s Archives of Pharmacology* updated its editorial guidelines in June 2020 to prevent future fraud publications in this journal, applying to all submitting authors. The respective text of the editorial guidelines is shown below:

Since the journal has experienced a rather extensive wave of fraud submissions of papers from paper mills and papers including fake data from external service laboratories, we had to extend the submission prerequisites to the following requests as stated in our Instructions for Authors:

https://www.springer.com/journal/210/submission-guidelines#Instructions%20for%20Authors_Important%20Submission%20Policy

- Request of institutional email addresses. At the minimum, at least the corresponding author should provide an institutional email address.
- Request of supplemental original source data (raw data, original data, individual data points) presented in tables and figures in a generally readable format. Excel files are preferred. Pdf and Prism files are acceptable as well. Supplemental data must be cited in the main text. These data will be made available to the reviewers and published if the paper is accepted.
- Request of supplemental immunoblot data. Specifically, full-length immunoblots with molecular mass markers are requested. Supplemental immunoblot data must be cited in the text. These data will be made available to the reviewers and published if the paper is accepted.
- Authors must include the following statement in the section “Authors Contributions”: The authors declare that all data were generated in-house and that no paper mill was used.

These measures are meant to protect the scientific integrity of your work and the scientific integrity of our journal.

Papers that do not follow these guidelines upon submission are administratively rejected but can be resubmitted when the formal requirements are met.

The new editorial policies have been in place now for more than 6 months. The overwhelming majority of authors abides to the rules and considers them as a quality criterion for their work and *Naunyn-Schmiedeberg’s Archives of Pharmacology*. To our delight, from several authors, we received very positive comments about the new editorial guidelines.

Only very few authors expressed concerns that original data could be “stolen” by peer reviewers. We take this concern extremely seriously and select only trustworthy reviewers with a proven track record and prefer referees whom the editors know personally. The Editor-in-Chief is not aware of a single case in which data from an author have been “stolen” by another scientist. We would react immediately if such scientific misconduct from the side of reviewers ever occurred. As a result of the paper mill attack, we are very reluctant at using author-proposed referees whom we do not know personally.

We realize that the updated editorial guidelines may appear “harsh” as compared to other scientific journals. However, due to the fact that *Naunyn-Schmiedeberg’s Archives of Pharmacology* had become an “attractive” target for paper mills, we had no other choice than to massively tighten our editorial guidelines to protect the integrity of our authors, the editors, the referees, the journal, and the publisher SpringerNature. In addition, we want to ensure that at its 150th anniversary in 2023, *Naunyn-Schmiedeberg’s Archives of Pharmacology* enjoys the status of a leading and rigorous pharmacological journal, promoting scientific integrity and quality. Interestingly, *Molecular Brain* also proposes that the inclusion of raw data along with the initial submission is the only solution to eradicate paper mills effectively (Miyakawa 2020).

The Editor-in-Chief, associate editors, reviewers, and the publisher SpringerNature do their very best to ensure that papers published in *Naunyn-Schmiedeberg’s Archives of Pharmacology* are based on honest science. We have weeded out a total of 50 fraudulent papers from the publication pipeline at various stages during the last year and will rigorously continue this path.

Finally, the Editor-in-Chief hopes that this editorial may be helpful to editors of other scientific journals how to handle the paper mill problem that has the malicious potential to cause a long-lasting and serious contamination of the scientific track record.
